# Cost-effectiveness of diagnosis and treatment of early gestational diabetes mellitus: economic evaluation of the TOBOGM study, an international multicenter randomized controlled trial

**DOI:** 10.1016/j.eclinm.2024.102610

**Published:** 2024-04-24

**Authors:** Mohammad M. Haque, W. Kathy Tannous, William H. Herman, Jincy Immanuel, William M. Hague, Helena Teede, Joanne Enticott, N. Wah Cheung, Emily Hibbert, Christopher J. Nolan, Michael J. Peek, Vincent W. Wong, Jeff R. Flack, Mark Mclean, Arianne Sweeting, Emily Gianatti, Alexandra Kautzky-Willer, Jürgen Harreiter, Viswanathan Mohan, Helena Backman, David Simmons, TOBOGM Consortium

**Affiliations:** a Translational Health Research Institute, Western Sydney University, Campbelltown, NSW, Australia; b School of Business, Western Sydney University, Parramatta, NSW, Australia; c Schools of Medicine and Public Health, University of Michigan, Ann Arbor, MI, United States; d School of Medicine, Western Sydney University, Campbelltown, NSW, Australia; e Robinson Research Institute, The University of Adelaide, Adelaide, SA, Australia; f Monash University, Melbourne, VIC, Australia; g Westmead Hospital and University of Sydney, Westmead, NSW, Australia; h Nepean Clinical School, University of Sydney and Nepean Hospital, Nepean, NSW, Australia; i Canberra Hospital, Canberra, ACT, Australia; j Australian National University, Canberra, ACT, Australia; k Liverpool Hospital, Liverpool and University of New South Wales, NSW, Australia; l Bankstown-Lidcombe Hospital, Bankstown, NSW, Australia; m Blacktown Hospital, Blacktown, NSW, Australia; n Department of Endocrinology, Royal Prince Alfred Hospital, Sydney, NSW, Australia; o Department of Endocrinology and Diabetes, Fiona Stanley and Fremantle Hospitals, Murdoch, WA, Australia; p Gender Medicine Unit, Division of Endocrinology and Metabolism, Department of Medicine III, Medical University of Vienna, Vienna, Austria; q Department of Medicine, Landesklinikum Scheibbs, Scheibbs, Austria; r Dr. Mohan's Diabetes Specialities Center and Madras Diabetes Research Foundation, Chennai, India; s Department of Obstetrics and Gynecology, Faculty of Medicine and Health, Orebro University, Orebro, Sweden

**Keywords:** Cost-effectiveness, Economic evaluation, First trimester, Gestational diabetes mellitus, Hyperglycemia, Neonatal intensive care, Pregnancy, Randomized controlled trial, Screening

## Abstract

**Background:**

A recently undertaken multicenter randomized controlled trial (RCT) “*Treatment Of BOoking Gestational diabetes Mellitus”* (TOBOGM: 2017–2022) found that the diagnosis and treatment of pregnant women with early gestational diabetes mellitus (GDM) improved pregnancy outcomes. Based on data from the trial, this study aimed to assess the cost-effectiveness of diagnosis and treatment of early GDM (from <20 weeks') among women with risk factors for hyperglycemia in pregnancy compared with usual care (no treatment until 24–28 weeks’) from a healthcare perspective.

**Methods:**

Participants’ healthcare resource utilization data were collected from their self-reported questionnaires and hospital records, and valued using the unit costs obtained from standard Australian national sources. Costs were reported in US dollars ($) using the purchasing power parity (PPP) estimates to facilitate comparison of costs across countries. Intention-to-treat (ITT) principle was followed. Missing cost data were replaced using multiple imputations. Bootstrapping method was used to estimate the uncertainty around mean cost difference and cost-effectiveness results. Bootstrapped cost–effect pairs were used to plot the cost-effectiveness (CE) plane and cost-effectiveness acceptability curve (CEAC).

**Findings:**

Diagnosis and treatment of early GDM was more effective and tended to be less costly, i.e., dominant (cost-saving) [−5.6% composite adverse pregnancy outcome (95% CI: −10.1%, −1.2%), −$1373 (95% CI: −$3,749, $642)] compared with usual care. Our findings were confirmed by both the CE plane (88% of the bootstrapped cost–effect pairs fall in the south-west quadrant), and CEAC (the probability of the intervention being cost-effective ranged from 84% at a willingness-to-pay (WTP) threshold value of $10,000–99% at a WTP threshold value of $100,000 per composite adverse pregnancy outcome prevented). Sub-group analyses demonstrated that diagnosis and treatment of early GDM among women in the higher glycemic range (fasting blood glucose 95–109 mg/dl [5.3–6.0 mmol/L], 1-h blood glucose ≥191 mg/dl [10.6 mmol/L] and/or 2-h blood glucose 162–199 mg/dl [9.0–11.0 mmol/L]) was more effective and less costly (dominant) [**−**7.8% composite adverse pregnancy outcome (95% CI: −14.6%, −0.9%), −$2795 (95% CI: −$6,638, −$533)]; the intervention was more effective and tended to be less costly [**−**8.9% composite adverse pregnancy outcome (95% CI: −15.1%, −2.6%), −$5548 (95% CI: −$16,740, $1547)] among women diagnosed before 14 weeks’ gestation as well.

**Interpretation:**

Our findings highlight the potential health and economic benefits from the diagnosis and treatment of early GDM among women with risk factors for hyperglycemia in pregnancy and supports its implementation. Long-term follow-up studies are recommended as a key future area of research to assess the potential long-term health benefits and economic consequences of the intervention.

**Funding:**

10.13039/501100000925National Health and Medical Research Council (grants 1104231 and 2009326), Region O¨rebro Research Committee (grants Dnr OLL-970566 and OLL-942177), Medical Scientific Fund of the Mayor of Vienna (project 15,205 and project 23,026), South Western Sydney Local Health District Academic Unit (grant 2016), and Western Sydney University Ainsworth Trust Grant (2019).

**Research in context**

**Evidence before this study**

We searched five electronic databases (including Medline and Embase) from the first available year to 31 December 2023 for economic evaluation or cost-effectiveness studies of treatment or management of gestational diabetes mellitus (GDM) published in English. The following search terms were used (economic∗ OR cost∗) AND (treat∗ OR manag∗) AND (gestational diabetes OR gdm). We identified two studies that examined and reported the cost-effectiveness of treating mild GDM. Moss et al. (2007) performed a cost-consequence analysis of the treatment of mild GDM based on the ACHOIS trial; using a decision analytic model, Ohno et al. (2011) found the treatment of mild GDM more effective and more costly. However, we found no previous studies that evaluated the cost-effectiveness of treating early GDM.

**Added value of this study**

A multicenter randomized controlled trial (RCT) “*Treatment Of BOoking Gestational diabetes Mellitus”* (TOBOGM) recently found that the diagnosis and treatment of pregnant women with early GDM improved pregnancy outcomes. This study is a prospective cost-effectiveness analysis of the TOBOGM study. Based on data from the trial, our study assessed the cost-effectiveness of diagnosis and treatment of early GDM (from <20 weeks') among women with risk factors for hyperglycemia in pregnancy compared with usual care (no treatment until 24–28 weeks') from a healthcare perspective. Participants' healthcare resource utilization data were collected from their self-reported questionnaires and hospital records. We found that diagnosing and treating early GDM was more effective and tended to be less costly, i.e., dominant (cost-saving) compared with usual care. Sub-group analyses suggested that greater cost-effectiveness was achieved among women in the higher glycemic range (fasting blood glucose 95–109 mg/dl [5.3–6.0 mmol/L], 1-h blood glucose ≥191 mg/dl [10.6 mmol/L] and/or 2-h blood glucose 162–199 mg/dl [9.0–11.0 mmol/L]); those diagnosed before 14 weeks’ gestation also tended to exhibit greater cost-effectiveness. Our study is the first to assess and add to the understanding of the cost-effectiveness of an intervention to diagnose and treat early GDM.

**Implications of all the available evidence**

There is published evidence for the cost-effectiveness of treating mild GDM from 24 weeks' gestation. Evidence on the cost-effectiveness of treating early GDM is needed to help guide national policies worldwide. Our results highlight the potential of diagnosing and treating early GDM cost-effectively among women with risk factors for hyperglycemia in pregnancy. Our study suggests that women with risk factors for hyperglycemia in pregnancy should be diagnosed and treated, preferably from before 14 weeks’ gestation and using higher band glucose criteria. Findings from our study will help healthcare providers and policymakers make better informed decisions to provide more cost-effective treatment strategies. Future studies should evaluate the long-term cost-effectiveness of treating early GDM to further assess the evidence base for its cost-effectiveness.

## Introduction

Gestational diabetes mellitus (GDM) is a common metabolic disorder, defined as hyperglycemia first identified during pregnancy and less than overt diabetes.^1^ It affects almost 15% of pregnancies across the world when the International Association of Diabetes and Pregnancy Study Groups (IADPSG) criteria are used for diagnosis.^2^ GDM is associated with adverse maternal and neonatal outcomes^3^, ^4^, ^5^ that can be effectively reduced by treatment beginning at 24–28 weeks’ gestation^6^^,^^7^ when the screening for this condition routinely occurs in most countries.^8^, ^9^, ^10^

The IADPSG recommends that pregnant women with risk factors for diabetes in pregnancy (DIP) complete one screening test at the first antenatal clinic booking appointment (usually by 14 weeks’) to identify undiagnosed pregestational diabetes.^8^^,^^11^ The options for assessment include an oral glucose tolerance test (OGTT), hemoglobin A1c (HbA1c), fasting or random plasma glucose testing which often identifies women with milder hyperglycemia than DIP.^1^ However, the glucose criteria for diagnosing milder hyperglycemia in early pregnancy (“early GDM”) remain unclear.^12^ Using various criteria, early GDM comprises 40–66% of GDM diagnoses,^13^, ^14^, ^15^ and is associated with more adverse pregnancy outcomes^16^, ^17^, ^18^ and insulin resistance^19^ than GDM diagnosed later in pregnancy.

There had been no previous large RCT of early GDM treatment^20^ (as opposed to early GDM screening^21^), and it remained unclear whether early treatment would be of benefit, or if the GDM diagnostic criteria applicable for 24–28 weeks' were appropriate for early pregnancy. Concerns had risen over an increased risk of greater special care nursery (SCN) or neonatal intensive care unit (NICU) admission and small for gestational age babies,^22^ an excessive reduction of fasting plasma glucose throughout the first trimester,^23^ and the frequent non-repeatability of an early OGTT GDM diagnosis at 24–28 weeks’.^24^ To assess if diagnosing and treating pregnant women with early GDM improved pregnancy outcomes, a multicenter randomized controlled trial (RCT) “*Treatment Of BOoking Gestational diabetes Mellitus”* (TOBOGM) was performed. The RCT found that the diagnosis and treatment of early GDM reduced the incidence of a composite of adverse pregnancy outcomes, NICU/SCN days, and severe perineal tears. Based on these results, a new standard of care was proposed.^25^

However, as healthcare resources are limited, a health-care intervention needs to be assessed for its impact on costs beyond clinical effectiveness. A health economic evaluation is highly recommended to be presented along with an RCT.^26^ Such analyses inform healthcare providers and policymakers about the relative cost-effectiveness of healthcare interventions in real-world settings and help them to decide which healthcare interventions to implement and/or reimburse, making the most efficient allocation of scarce healthcare resources to improve health outcomes.^27^ Based on data from the TOBOGM trial, this study aimed to assess the cost-effectiveness of diagnosis and treatment of early GDM (from <20 weeks') among women with risk factors for hyperglycemia in pregnancy compared with usual care (no treatment until 24–28 weeks’) from a healthcare perspective.

## Methods

### Brief description of the trial

The study design of the TOBOGM RCT has been described in detail elsewhere.^25^ In brief, TOBOGM was an RCT conducted in 17 hospitals across four countries (Australia, Austria, India, and Sweden) between 2017 and 2022. The study protocol^28^ was approved by South Western Sydney Local Health District Human Research Ethics Committee (reference, HREC/15/LPOOL/551). All participants provided their written informed consent before the start of the study. The RCT was registered with the Australian New Zealand Clinical Trials Registry (Identifier ACTRN12616000924459).^29^

Adult (≥18 years of age) women with a singleton pregnancy at 4–19^+6^ weeks', and at least one risk factor for hyperglycemia in pregnancy^10^ attending their first antenatal visits at a collaborating antenatal clinic or hospital were considered eligible for inclusion and invited to participate in the trial. Consecutive eligible women willing to participate that provided written informed consent were referred for a 2-h 75 g OGTT before 20 weeks’. Those with recognized pre-existing diabetes, fasting blood glucose (FBG) ≥ 110 mg/dl (6.1 mmol/L) and/or 2-h blood glucose (2HBG) ≥ 200 mg/dl (11.1 mmol/L), or major active medical disorders were excluded from the RCT.

Participants diagnosed with GDM according to World Health Organization (WHO) 2013 diagnostic criteria (FBG ≥92 mg/dl [5.1 mmol/L], 1-h blood glucose (1HBG) ≥ 180 mg/dl [10.0 mmol/L], and/or 2HBG ≥153 mg/dl [8.5 mmol/L])^1^ before 20 weeks' were randomly allocated. The allocation was in 1:1 ratio to either immediate treatment for early GDM (early management group) or deferred/no treatment (usual care group), depending on the results of a repeat OGTT performed at 24–28 weeks'. Randomization by minimization was performed by an electronic randomization program (Techtonic, UK) and stratified by the site and two glycemic ranges, lower (FBG 92–94 mg/dl [5.1–5.2 mmol/L], 1HBG 180–190 mg/dl [10.0–10.5 mmol/L] and/or 2HBG 153–161 mg/dl [8.5–8. 9 mmol/L]) and higher (FBG 95–109 mg/dl [5.3–6.0 mmol/L], 1HBG ≥191 mg/dl [10.6 mmol/L] and/or 2HBG 162–199 mg/dl [9.0–11.0 mmol/L]). These glycemic ranges were based upon 1.75 and 2.0-fold risks of adverse pregnancy outcomes at 24–28 weeks’ as per the Hyperglycemia and Adverse Pregnancy Outcome (HAPO) study.^3^^,^^11^ All participants, hospitals and trial staff were blinded to OGTT results to prevent management bias, unless the results were FBG ≥110 mg/dl (6.1 mmol/L) and/or 2HBG ≥200 mg/dl (11.1 mmol/L), in which case women were excluded from the study and treated.

Women in the usual care group received routine antenatal care from obstetricians and/or midwives as per the local guidelines. They did not receive treatment for GDM unless they were diagnosed with GDM later at 24–28 weeks’ using the WHO 2013 criteria.^1^

Women in the early management group received treatment for early GDM, in addition to standard antenatal care, following the consensus GDM management guidelines.^28^ This consisted of at least one session with a diabetes educator about GDM, one session with a qualified dietitian advising on a sustainable healthy dietary pattern, and instructions on self-monitoring of blood glucose (SMBG). Participants were asked to perform SMBG four times a day and to attend clinics on a regular basis to review the results. Pharmacological treatment (insulin and/or metformin) was initiated and intensified according to the standard local practice for pregnant women with FBG ≥95 mg/dl (5.3 mmol/L), 2HBG ≥126 mg/dl (7.0 mmol/L), or two high blood glucose values within seven days with no obvious cause.^6^^,^^7^

The clinical outcome measure of the RCT was a composite of adverse pregnancy outcomes, which included pre-term birth <37 weeks’, birthweight ≥4.5 kg, shoulder dystocia, birth trauma, neonatal respiratory distress syndrome, phototherapy requirement for jaundice/hyperbilirubinemia, and/or stillbirth/neonatal death. Laboratory tests and neonatal anthropometric measurements were taken as per standardized methodology as described elsewhere.^25^^,^^28^^,^^29^ There was a reduction of adverse pregnancy outcome in the early management group compared to the control group [adjusted risk difference: −5.6%; 95% confidence interval (CI): −10.1%, −1.2%].^25^ Adjusted risk difference in composite adverse pregnancy outcome with 95% CI between the intervention and control groups were determined with the use of mixed-effects models, adjusting for six prespecified factors: age, ethnicity, pre-pregnancy body mass index (BMI), primigravidity, education, and current smoking status.^25^

### Cost data collection and valuation

Costs were measured from a healthcare perspective from randomization (<20 weeks') until postnatal hospital discharge of the mother and newborn, which was the time horizon for economic evaluation. Only direct healthcare costs were considered, which included costs related to the diagnosis for early (<20 weeks') and standard (24–28 weeks’) GDM, primary healthcare (general practitioner, midwife), secondary healthcare (endocrinologist, obstetrician/maternal–fetal medicine specialist, miscellaneous healthcare providers), blood tests, laboratory diagnostic tests (ultrasound, fetal non-stress test), emergency department attendance, maternal hospitalization, delivery of birth, neonatal hospitalization to SCN/NICU, allied healthcare (dietician, diabetes educator), supplies for SMBG and medication.

The number of eligible women for the trial derived from the CONSORT diagram^25^ (Appendix A. Supplementary data: Fig. S1) was considered in estimating the cost for early GDM diagnosis. The cost of an additional 3.2 OGTT for each woman with early GDM identified was included as the cost for early GDM diagnosis in the early management group but not in the usual care group (Appendix A. Supplementary data: Attachment S1). Participants' healthcare resource utilization was assessed using the data extracted from self-reported questionnaires completed at 24–28 weeks' and 35–37 weeks'. The questionnaires collected information about the participants’ type of healthcare service utilized, number of clinical visits made, type (name, composition, and dose) and the number of treatment days of medication taken. Additional resource utilization data concerning the maternal hospitalization, delivery of birth, and neonatal hospitalization to SCN/NICU were collected from the hospital administrative records.

Costs were estimated retrospectively using a bottom-up micro-costing approach by multiplying the reported quantity of each resource item consumed by the respective standard Australian average unit cost. Health care resources were valued using the unit costs obtained from standard Australian national sources.^30^, ^31^, ^32^ Unit costs for medications were obtained from the Pharmaceutical Benefits Scheme (PBS).^30^ Unit costs for general practitioner, midwife, endocrinologist, obstetrician/maternal–fetal medicine specialist, miscellaneous healthcare providers, dietician, diabetes educator, blood test, ultrasound, and fetal non-stress test were derived from the Medicare Benefits Schedule (MBS).^31^ Emergency department attendance, maternal hospitalization, mode of birth, and SCN/NICU admission were valued using the Australian Refined Diagnosis Related Group (AR-DRG) classified cost weights sourced from the National Hospital Cost Data Collection (NHCDC) Public Sector Report (2019–20).^32^

SMBG cost involved three components: the glucometer, blood glucose test strips and lancets. The cost for blood glucose test strips covered by the Australian National Diabetes Service Scheme (NDSS) was in consideration of this study. Women in the early management group (and those diagnosed with GDM later at 24–28 weeks’ in the usual care group) were assumed to use four test strips per day based on the per-protocol testing frequencies. Details of unit costs and healthcare services utilization are provided in Appendix A. Supplementary data Tables S1–S6.

All costs were initially collected in 2022 Australian dollars (A$). Prices were adjusted for inflation when necessary, using a national inflation factor for medical and hospital services of the Australian consumer price index.^33^ Costs were finally converted into US dollars ($) using the purchasing power parity (PPP) estimates^34^ to facilitate comparison of costs across countries. Costs were not discounted as the time horizon of the analysis was less than one year.

### Statistical analysis

The cost-effectiveness analysis was performed on an intention-to-treat (ITT) basis. Following careful consideration of the pattern of missing cost data, they were assumed to be missing at random and imputed based on multiple imputation by chained equations as the most suitable option for our missing data patterns.^35^ Ten complete data sets were generated for each set of missing data. Pooled estimates of costs were calculated from the generated datasets using Rubin's rules.^35^, ^36^ More details are presented in Appendix A. Supplementary Data: Attachment S2.

In an economic evaluation, an intervention is considered cost-effective compared to its comparator if: (i) it is more effective and less costly (i.e., dominant); or (ii) it is more effective and more costly, but the society is willing to pay for its additional cost per additional unit of effect, i.e., incremental cost-effectiveness ratio (ICER); or (c) it is less effective and less costly, but the ICER of its comparator is not considered worth paying by the decision makers.^26^ ICER is calculated when one alternative is more (less) effective and more (less) costly compared with another, i.e., neither treatment arm dominates. If the intervention arm is “dominant” (i.e., more effective and less costly), an ICER is not calculated.

The cost-effectiveness of the diagnosis and treatment of early GDM among women with risk factors for hyperglycemia in pregnancy was assessed by comparing the costs and effects incurred in the early management group with those of the usual care group and estimating the ICER if appropriate. Because of the positive skewness in the non-normal distribution of cost data, a non-parametric bootstrapping method (bias-corrected and accelerated) with 1000 replications was used to estimate 95% CI around the mean cost difference.

The bootstrap process was also used to evaluate the overall uncertainty of cost-effectiveness of the intervention. Bootstrapped incremental cost–effect pairs were plotted on a cost-effectiveness (CE) plane to visually represent the difference in costs (plotted on y-axis) and outcomes (plotted on x-axis) between the treatment alternatives, resulting in four quadrants. Each quadrant of the CE plane indicates whether the intervention is less effective-more costly, less effective-less costly, more effective-less costly, or more effective-more costly compared with usual care. A cost-effectiveness acceptability curve (CEAC) was also constructed which shows the probability of the intervention being cost-effective compared with usual care over a range of possible values for the decision-maker's willingness-to-pay (WTP) thresholds per composite adverse pregnancy outcome prevented. As no formal WTP threshold exists for any of the participating countries, the commonly quoted thresholds of $10,000 (minimum) and $100,000 (maximum) were used as representing good value for the money. However, it depends on the decision makers what they are willing to pay per composite adverse pregnancy outcome prevented.

All analyses were performed using Stata 17.0. Statistical significance was considered at p < 0.05.

### Sub-group analyses

Two prespecified sub-group analyses were performed. The first analysis was based on the glycemic range at randomization, i.e., higher glycemic range versus lower glycemic range. The second analysis was conducted by the initial OGTT timing at trial entry using the following classification: <14 weeks' versus 14–19^+6^ weeks’.

### Sensitivity analysis

In the sensitivity analysis, economic analysis was performed according to the per-protocol principle, and restricted solely to participants with complete healthcare resource use and outcome data (i.e., *complete cases*) to assess the potential bias due to missing values.

### Role of the funding source

The funder(s) of the study had no role in the study design, data collection, data analysis, data interpretation, writing of the manuscript, or the decision to submit the manuscript for publication.

## Results

### Participants

Of the 43,721 women assessed for entry into the RCT, 3681 undertook an early OGTT of whom 802 women were diagnosed with early GDM and randomized. Of these, 396 (49.4%) were assigned to the usual care group and 406 (50.6%) to the early management group. The final study sample consisted of 793 participants (393 usual care vs 400 early management) after nine participants were excluded because of early pregnancy loss. The flowchart and baseline characteristics (including age, ethnicity, body mass index, education, medical history, primigravida, smoking) of the study participants have been reported previously (Appendix A. Supplementary data Fig. S1).^25^ Participants’ demographic characteristics at baseline were similar in the two groups. Complete healthcare cost data were available for 742 (93.6%) participants.

### Costs

Table 1 provides an overview of the mean total healthcare cost (with a breakdown by the cost category) and mean total and disaggregated cost differences per participant by the study group. In both groups, most of the healthcare cost were attributable to the birthing cost, followed by the maternal hospitalization, NICU, and SCN costs. Mean costs were higher in the early management group than in the usual care group for all cost categories except the midwife, miscellaneous healthcare providers, delivery of birth, NICU, and SCN. Significant differences were observed between the two groups only in mean costs related to the early GDM diagnosis, SMBG, endocrinologist, obstetrician/maternal–fetal medicine specialist, midwife, dietician, diabetes educator, emergency department and medication. The mean total healthcare cost per participant in the early management group ($12,121 SE = $587) was lower by 10.2% than in the usual care group ($13,494 SE = $932). However, this difference in mean total healthcare costs [−$1373 (95% CI: −$3,749, $642)] was not statistically significant.

**Table 1:** Mean healthcare cost (SE) and mean cost differences (95% CI) per participant by cost category for the early management and usual care groups.

Cost category	Early management group (n = 400) $ (SE)	Usual care group (n = 393) $ (SE)	Cost difference $ (95% CI)
Early GDM diagnosis approach^a^	38.70 (0.22)	0	**38.70 (37.73, 39.67)**
Self-monitoring of blood-glucose	69.14 (0.48)	39.07 (1.73)	**30.07 (26.93, 33.85)**
General practitioner	31.63 (2.97)	31.56 (3.34)	0.07 (−9.16, 7.96)
Endocrinologist	214.24 (14.07)	91.0 (7.25)	**123.24 (91.84, 155.10)**
Obstetrician/Maternal-fetal medicine specialist	353.52 (11.17)	276.41 (10.12)	**77.11 (48.40, 105.81)**
Midwife	115.55 (4.54)	138.13 (5.16)	**−22.58 (−37.11, −10.36)**
Miscellaneous healthcare providers^b^	151.98 (16.88)	158.14 (17.40)	−6.16 (−55.93, 38.18)
Dietician	55.79 (1.58)	41.28 (1.77)	**14.51 (9.84, 18.75)**
Diabetes educator	128.13 (3.98)	71.39 (3.24)	**56.74 (47.49, 67.83)**
Ultrasound	204.68 (9.93)	179.74 (8.39)	24.94 (−1.11, 49.55)
Fetal non-stress test	0.54 (0.24)	0.20 (0.10)	0.34 (−0.06, 0.99)
Blood test	19.20 (1.29)	17.88 (1.72)	1.32 (−3.23, 5.19)
Emergency department	108.96 (19.21)	69.63 (11.39)	**39.33 (1.56, 87.48)**
Maternal hospital admission	1466.67 (33.21)	1437.12 (33.39)	29.55 (−65.39, 125.02)
Delivery of birth	6601.31 (152.06)	6854.26 (152.42)	−252.95 (−659.98, 170.46)
Neonatal intensive care unit	859.47 (498.03)	2186.35 (858.65)	−1326.88 (−3616.92, 397.24)
Special care nursery	1521.44 (256.73)	1811.03 (291.85)	−289.59 (−1052.58, 394.88)
Medication	179.63 (10.96)	90.72 (6.77)	**88.91 (63.77, 113.74)**
Total healthcare	12,120.58 (586.63)	13,493.91 (931.68)	−1373.33 (−3748.79, 641.94)

Note: Statistical significance (p < 0.05) denoted by bold highlight. Abbreviations: $ United States dollar; 95% CI 95% Confidence interval “bias corrected and accelerated”; GDM Gestational diabetes mellitus; SE Standard error.

a Appendix A. Supplementary data: Attachment S1.

b Include anesthesiologist, birth/day/women's assessment unit, cardiologist, chronic disease nurse, consultant physician (dental, renal, thyroid), hematologist, mental health nurse, mental health service, neurologist, ophthalmologist, orthopedic, pediatrician, physiotherapist, podiatrist, pre-admission, preeclampsia day stay, psychologist, social worker, and urologist. Statistical significance denoted by bold highlight.

### Cost-effectiveness analysis

**Fig. 1:**
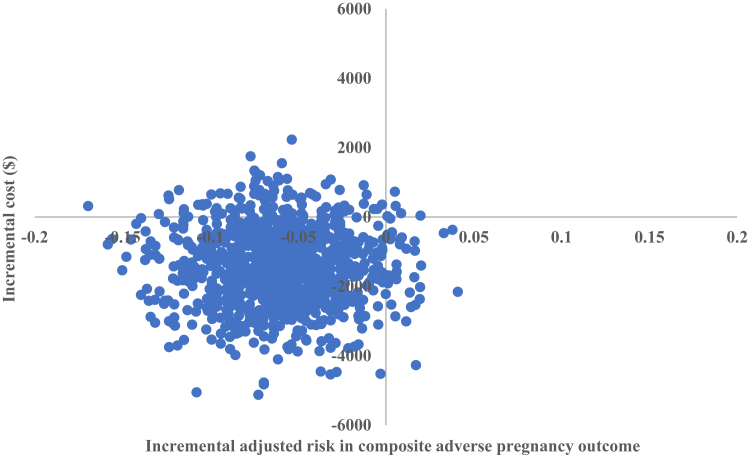
**Cost-effectiveness (CE) plane demonstrating the probability of cost-effectiveness (using 1000 bootstrap simulations) for early GDM diagnosis and treatment among women with risk factors for hyperglycemia in pregnancy compared with usual care**. 0.5% of the bootstrapped cost–effect pairs fall in the north-east quadrant (less effective, more costly); 3.8% of the bootstrapped cost–effect pairs fall in the south-east quadrant (less effective, less costly); 87.8% of the bootstrapped cost–effect pairs fall in the south-west quadrant (more effective, less costly); 7.9% of the bootstrapped cost–effect pairs fall in the north-west quadrant (more effective, more costly). Abbreviations: $ US dollars; GDM Gestational diabetes mellitus.

**Fig. 2:**
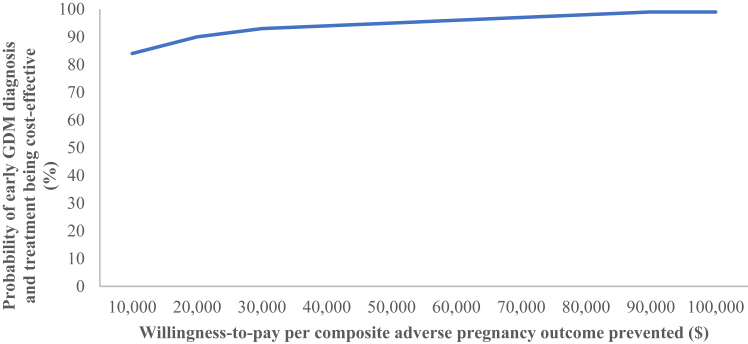
**Cost-effectiveness acceptability curve (CEAC) showing the probability of early GDM diagnosis and treatment being cost-effective among women with risk factors for hyperglycemia in pregnancy for different ceilings of willingness-to-pay (WTP) compared with usual care for composite adverse pregnancy outcome**. Abbreviations: $ US dollars; GDM Gestational diabetes mellitus.

The within-trial cost-effectiveness analysis is reported in Table 2. Diagnosis and treatment of early GDM among women with risk factors for hyperglycemia in pregnancy was more effective and tended to be less costly, i.e., dominant (cost-saving) compared with usual care. As the CE plane shows (Fig. 1), 88% of the bootstrapped cost–effect pairs fall in the south-west quadrant, indicating that the intervention, on average, was likely to be more effective and less costly, i.e., dominant (cost-saving) compared with usual care. The CEAC (Fig. 2) confirms the findings that the intervention had a high probability of being cost-effective, ranging from 84% at a WTP threshold value of $10,000–99% at a WTP threshold value of $100,000 per composite adverse pregnancy outcome prevented. Even if healthcare decision-makers were not willing to pay anything for the prevention of one composite adverse pregnancy outcome (i.e., WTP = $0/per decrease in composite adverse pregnancy outcome), the intervention was still 79% likely to be cost-effective compared with usual care.

**Table 2:** Cost-effectiveness analysis of the diagnosis and treatment of early GDM among women with risk factors for hyperglycemia in pregnancy.

Analysis	Sample size (n)		Adjusted risk difference in composite adverse pregnancy outcome^a^ ΔE (95% CI) %	Incremental cost ΔC (95% CI) $	ICER ($ per composite adverse pregnancy outcome prevented)
	Early management group	Usual care group			
Base case analysis	400	393	**−5.6 (−10.1, −1.2)**	−1373 (−3749, 642)	Dominant (cost saving)
**Sub-group analysis–Glycemic range**^b^					
Higher glycemic range^c^	229	231	**−7.8 (−14.6, −0.9)**	**−2795 (−6638, −533)**	Dominant (cost saving)
Lower glycemic range^d^	171	162	−2.5 (−10.4, 5.5)	646 (−1864, 4326)	Not applicable^e^
**Sub-group analysis—Timing of oral glucose tolerance test**					
<14 weeks' gestation	105	79	**−8.9 (−15.1, −2.6)**	−5548 (−16740, 1547)	Dominant (cost saving)
14–19^+6^ weeks' gestation	295	314	−5.0 (−11.6, 1.6)	−409 (−2100, 765)	Dominant (cost saving)
Sensitivity analysis (complete case analysis)	376	366	**−5.7 (−9.3, −2.1)**	−1465 (−4152, 597)	Dominant (cost saving)

Note: Statistical significance (p < 0.05) denoted by bold highlight. Abbreviations: $ United States dollar; ΔC Change in cost; ΔE Change in effect; 1HBG 1-h blood glucose; 2HBG 2-h blood glucose; 95% CI: 95% Confidence interval “bias corrected and accelerated”; FBG Fasting blood glucose; GDM Gestational diabetes mellitus; ICER Incremental cost-effectiveness ratio.

a Composite adverse pregnancy outcome included pre-term birth <37 weeks' gestation, birthweight ≥4.5 kg, shoulder dystocia, birth trauma, neonatal respiratory distress syndrome, phototherapy requirement for jaundice/hyperbilirubinemia, and/or stillbirth/neonatal death. Adjusted risk difference in composite adverse pregnancy outcome with 95% CI between the intervention and control groups were determined with the use of mixed-effects models, adjusting for six prespecified factors: age, ethnicity, pre-pregnancy body mass index (BMI), primigravidity, education, and current smoking status.^25^

b Glycemic ranges were based upon 1.75 and 2.0-fold risks of adverse pregnancy outcomes at 24–28 weeks' gestation as per the Hyperglycemia and Adverse Pregnancy Outcome (HAPO) study.^3^^,^^11^

c Higher glycemic range: FBG 95–109 mg/dl (5.3–6.0 mmol/L), 1HBG ≥191 mg/dl (10.6 mmol/L) and/or 2HBG 162–199 mg/dl (9.0–11.0 mmol/L).

d Lower glycemic range: FBG 92–94 mg/dl (5.1–5.2 mmol/L), 1HBG 180–190 mg/dl (10.0–10.5 mmol/L) and/or 2HBG 153–161 mg/dl (8.5–8.9 mmol/L).

e ICER was not calculated since the diagnosis and treatment of early GDM was more likely to be harmful for neonates of mothers in the lower glycemic range due to a possibility of an increased risk of small-for-gestational-age infants. Statistical significance denoted by bold highlight.

### Sub-group analyses

The mean difference between the costs and effects in sub-groups by the glycemic range and timing of early OGTT are presented in Table 2. Sub-group analyses demonstrated that diagnosis and treatment of early GDM was more effective and less costly [−7.8% composite adverse pregnancy outcome (95% CI: −14.6%, −0.9%), −$2795 (95% CI: −$6,638, −$533)] among women in the higher glycemic range; the intervention was more effective and tended to be less costly [−8.9% composite adverse pregnancy outcome (95% CI: −15.1%, −2.6%), −$5548 (95% CI: −$16,740, $1547)] among women diagnosed before 14 weeks’ as well. Diagnosis and treatment of early GDM tended to be modestly cost saving [−5.0% composite adverse pregnancy outcome (95% CI: −11.6%, 1.6%), −$409 (95% CI: −$2,100, $765)] for the OGTT 14–19^+6^ weeks-only sample, while the lower glycemic range-only sample tended to be cost-incremental [−2.5% composite adverse pregnancy outcome (95% CI: −10.4%, 5.5%), $646 (95% CI: −$1,864, $4326)].

### Sensitivity analysis

The sensitivity analysis (including only the complete cases) showed similar results to those in the base case analysis (Table 2) indicating that the overall conclusions of our study would not change if the missing data were not imputed. The intervention tended to remain dominant (cost-saving) with a high probability of lower mean cost per participant and improved clinical outcomes.

## Discussion

This study evaluated the cost-effectiveness of diagnosis and treatment of early GDM among women with risk factors for hyperglycemia in pregnancy compared with usual care from a healthcare perspective based on data from the TOBOGM trial. Overall, our findings suggested that the intervention reduced composite adverse pregnancy outcomes [−5.6% (95% CI: −10.1%, −1.2%)] and was less costly [−$1373 (95% CI: −$3,749, $642)] compared with usual care. The difference in costs of total healthcare utilization incurred between the early management and control groups in the primary analysis was not statistically significant. However, it is recommended in health economics literature to consider the joint density of cost and effect differences, instead of separate and sequential hypothesis tests concerning cost and effect differences, while making the decision to estimate incremental cost-effectiveness.^37^^,^^38^ Our results (CE plane and CEAC) suggested that the cost and effect differences considered jointly produce a high probability of the intervention being more effective and less costly, i.e., dominant (cost saving). The early management group, on average, had a modestly higher utilization in most healthcare services (except the midwife, miscellaneous healthcare providers, delivery of birth, and NICU/SCN) than the usual care group. However, the higher costs incurred by the early management group compared with the usual care group were entirely offset by the cost savings in other healthcare services, mainly through the reduction in complicated deliveries, SCN/NICU admissions [−3% (95% CI: −7%, 0%)], and a shorter duration of SCN/NICU stay [−0.8 bed days (95% CI −1.3, −0.3)]. These results remained robust in the additional sensitivity analysis using a complete case analysis. The complementary sub-group analyses demonstrated that the diagnosis and treatment of early GDM among women in the higher glycemic range was more effective and less costly (dominant) [−7.8% composite adverse pregnancy outcome (95% CI: −14.6%, −0.9%), −$2795 (95% CI: −$6,638, −$533)]; the intervention was also more effective and tended to be less costly among women diagnosed before 14 weeks’ [−8.9% composite adverse pregnancy outcome (95% CI: −15.1%, −2.6%), −$5548 (95% CI: −$16,740, $1547)].

Our study demonstrated that undiagnosed and untreated early GDM causes additional costs to the healthcare system, especially for costs associated with the birth and neonatal hospitalization. Mothers with untreated early GDM were more likely to have a complicated delivery (vaginal birth with instrument or 3rd/4th degree tear). Such modes of birth are more costly than the normal vaginal birth, eventually contributing to the higher antenatal inpatient expenditure for women with untreated early GDM. Neonates born to mothers with untreated early GDM were non-significantly more likely to be admitted to SCN/NICU than those born to mothers with treated early GDM. The greater number of SCN/NICU admissions for babies of mothers with untreated early GDM could be largely explained by the higher percentage of neonatal respiratory distress. Our study suggested that the diagnosis and treatment of early GDM among women with risk factors for hyperglycemia in pregnancy could produce cost savings to the healthcare system especially through reductions in the number of complicated deliveries, SCN/NICU admission, and SCN/NICU daily costs (via shorter LOS).

There are no other previous cost-effectiveness studies of early GDM treatment intervention. Previous cost-effectiveness studies concerning the treatment of mild GDM at 24–28 weeks’ showed that the interventions were cost-effective in other countries and populations (Australia, UK, and USA).^39^^,^^40^ Moss et al. performed a cost-consequence analysis of the treatment of mild GDM based on the Australian Carbohydrate Intolerance Study in Pregnant Women (ACHOIS) trial and reported its cost-effectiveness at A$27,503 per additional serious perinatal complication prevented, A$2988 per discounted life-year gained, and A$60,506 per perinatal death prevented.^39^ Using a decision analytic model, Ohno et al. found the treatment of mild GDM in the USA to be more effective and more costly and estimated its cost-effectiveness at $20,412 per quality-adjusted life-year (QALY) gained.^40^ Our study was the first to assess the cost-effectiveness of an intervention to diagnose and treat early GDM; the health outcomes considered in our cost-effectiveness analysis differ from those used in the previous studies.^39^^,^^40^ Therefore, our findings cannot be directly compared with the results from these studies. However, the high costs associated with the neonatal hospitalization and delivery of birth in our study are consistent with those of previous studies.

This study has several strengths. It is a prospective and comprehensive economic evaluation of an international, multicenter RCT performed in a real-world setting with a usual care control condition and objectively measured clinical outcomes. The large number of participants from ethnically diverse populations included in each arm, the success of randomization as indicated by the similar baseline characteristics within treatment groups, and the high retention rate, all strengthened the quality and quantity of the resource utilization data.

Our economic analysis is based on the patient-level economic data directly derived during the study; the data therefore reflected the actual healthcare resource utilization, thus making the results more reliable. Resource utilization data concerning the delivery of birth and neonatal hospitalization (the biggest cost contributors) were collected from reliable hospital administrative records.

Another important strength of the study is its use of appropriate statistical and econometric techniques such as multiple imputation, bootstrapping, sub-group analyses, and sensitivity analysis. Furthermore, the evaluation has been performed from a healthcare perspective, the perspective preferred by health insurance companies which may consider reimbursing the direct medical costs incurred by the early diagnosis and treatment program.

This study has some limitations which should be considered while interpreting the results. Firstly, most healthcare resource utilization data (except the maternal hospitalization, delivery of birth and neonatal hospitalization) have been collected intermittently and retrospectively using the participants’ self-reported questionnaires. This may have caused social desirability and recall bias and affected the accuracy of the cost estimates. However, the effect of these biases (under- or overestimation of resource usage reporting) would be minimal on our study findings as this approach was applied to both groups. Secondly, the study did not use unit cost data of all the participating countries because of their limited availability; unit costs were based on the Australian costing data. This limitation was addressed by converting the costs into US dollars using the PPP estimates to facilitate comparison of costs across countries.

Thirdly, missing data were addressed by the multiple imputation technique. Multiple imputation is based on the assumption that unobserved data partly depend on the observed data (e.g. available costs), that cannot be fully tested. However, only 6.4% of all cost data were imputed. Therefore, the effect of any potential error with cost estimate on our cost-effectiveness analysis would be minimal. Fourthly, participants for this study were recruited from the populations at high-risk for GDM, and may not be representative of the wider population of pregnant women. In addition, antenatal care and diabetes management protocols differ across countries. Therefore, our findings may not be widely generalizable to all pregnant women and all country settings.

Fifthly, our study considered only the direct healthcare costs consistent with a healthcare perspective; indirect and/or direct non-medical costs were not included. Therefore, our findings may not have sufficiently addressed the local policy requirements of the participating countries (e.g. India has a multi-payer universal health care model that is paid for jointly by the government-funded public hospitals and public and government regulated private health insurances). However, the indirect and/or direct non-medical costs costs were not substantial in the previous cost-effectiveness studies of GDM management.^39^^,^^40^ Future research could be conducted using a broader societal perspective.

Sixthly, our cost-effectiveness analysis did not consider QALYs as a measure of health outcome. The main trial was designed to collect health-related quality of life (QoL) data; however, we had a very low completion rate (35%) on the post-partum EQ-5D questionnaire. The high level of missing post-natal EQ-5D data made it difficult to reliably impute them and estimate the incremental QALY (as the robustness of an imputation method declines with the increasing level of missing data, adversely affecting the validity and reliability of an analysis). This may affect the consideration of the intervention by policy makers of some countries (e.g. the UK), where ICER is required to be expressed in terms of cost per QALY for local decision-making. However, the composite adverse pregnancy outcome considered in our cost-effectiveness analysis is a useful and acceptable health outcome measure in assessing the effectiveness of interventions for hyperglycemia in pregnancy.^6^^,^^7^^,^^39^^,^^40^ Future research could be conducted including QALY as a health outcome measure.

Seventhly, the sample size of participants for some sites (e.g. Austria, India) was not adequate to perform sub-group analyses for each participating country. There is an increased risk of type II errors (i.e., inability to reliably detect meaningful true effects with a reasonable level of confidence when they exist) with underpowered analyses. Such threat to statistical validity undermines the accuracy of findings and compromise the overall reliability and validity of statistical analyses. Given the variations in health systems of the participating countries and decision making tends to be local, our pooled findings as currently presented may not be sufficiently useful to inform local policy decisions in any of the collaborating countries. Future research could be performed addressing this.

Finally, our study was limited by its short time horizon. Only the short-term impact of the intervention on the clinical outcomes and healthcare costs was explored. However, the intervention may have favourable broader impact on the health outcomes of both mother and child, and healthcare costs beyond the pregnancy period over a longer term (e.g. lifetime horizon). Therefore, further research with a longer follow-up is recommended to ascertain the longer-term cost-effectiveness of this intervention.

This study has important implications for healthcare providers and policymakers worldwide. Our findings highlight the potential economic benefits from the diagnosis and treatment of early GDM among women with risk factors for hyperglycemia in pregnancy to prevent pregnancy complications. The results of our study indicate that the intervention was more effective and tended to be less costly, i.e., dominant (cost saving) in comparison with usual care, primarily due to the large reduction in costs associated with the complicated delivery of birth and neonatal hospitalization to SCN/NICU. Greater cost-effectiveness was demonstrated among women within the higher glucose range; those diagnosed before 14 weeks’ also tended to demonstrate the same. The study did not attempt to estimate or incorporate potential long-term health benefits and economic consequences of the intervention. Long-term follow-up studies are therefore suggested as a key future area of research to further assess the evidence base on the cost-effectiveness of diagnosis and treatment of early GDM. Based on these results, our study supports for implementation of the diagnosis and treatment of early GDM among women with risk factors for hyperglycemia in pregnancy.

## Contributors

DS conceived, designed, and supervised the clinical trial. DS was the principal investigator of the clinical study and contributed to the interpretation of clinical findings. MMH designed the cost-effectiveness study, collected the economic data, and performed statistical analyses of the economic data with major contributions of KT. MMH prepared the initial draft version of the manuscript. MMH, KT, WHH, and DS contributed to the interpretation of the data. All authors read, thoroughly revised, critically reviewed, and corrected draft versions of the manuscript and approved the final version of the manuscript for publication. DS and KT are the guarantors of this work and, as such, had full access to all the data in the study and takes responsibility for the integrity of the data and the accuracy of the data analysis.

## Data sharing statement

There are no unpublished data from the study. All relevant cost data are included in this article (and its Supplementary Files). Complete de-identified patient dataset generated or analyzed in this study are available from the corresponding author according to institutional policies and upon reasonable request by providing a detailed ethics-approved protocol for the proposed study, information about tssources to carry out the study, approval by the TOBOGM Trial management Group, and a completed data sharing agreement.

## Declaration of interests

WHH reports participation on Merck Sharp & Dohme Board and Rivus Pharmaceuticals Board. DS reports Presidency of the Australasian Diabetes in Pregnancy Society. All otherauthor(s) have no potential conflict of interests to report.
