# Supplementary material for: Cost-effectiveness of diagnosis and treatment of early gestational diabetes mellitus: economic evaluation of the TOBOGM study, an international multicenter randomized controlled trial
Source: eClinicalMedicine. 2024 Apr 24;71:102610. doi: 10.1016/j.eclinm.2024.102610 (PMC11133791; doi:10.1016/j.eclinm.2024.102610)
Supplement: Appendix [file mmc1.docx]

**Cost-effectiveness of diagnosis and treatment of early gestational diabetes mellitus: economic evaluation of the TOBOGM study, an international multicenter randomized controlled trial**

Mohammad M. Haque, Kathy Tannous, William H. Herman, Jincy Immanuel, William M. Hague, Helena Teede, Joanne Enticott, N. Wah Cheung, Emily Hibbert, Christopher J. Nolan, Michael J. Peek, Vincent W. Wong, Jeff R. Flack, Mark Mclean, Arianne Sweeting, Emily Gianatti, Alexandra Kautzky-Willer, Jürgen Harreiter, Viswanathan Mohan, Helena Backman, and David Simmons on behalf of the TOBOGM consortium*.*

**Appendix A. Caption for Supplementary Material**

**Table of contents**

**Figure S1:** CONSORT diagram of screening, randomization, and follow-up of the TOBOGM trial………….p-2

**Attachment S1:** Cost estimation for early gestational diabetes mellitus (GDM) diagnosis………………………………………………………………………………….……………………..p-3

**Attachment S2:** Missing data and multiple imputation……………….………………………………………..p-4

**Table S1:** Unit cost of primary, secondary, and allied healthcare services utilized in the main analysis………p-5

**Table S2:** Unit of healthcare service utilization per participant by cost category for the early management and usual care groups for complete case participants………………………………………………………………..p-7

**Table S3:** Number and percentage of NICU and SCN admission by the early management and usual care groups for complete case participants…………………………………………………………………………………...p-8

**Table S4:** Length of stay of NICU and SCN admission by the early management and usual care groups for complete case participants……………………………………………………………………………………….p-9

**Table S5:** Number and percentage of mode of birth by the early management and usual care groups for complete case participants……………………………………………………………………………………………..…p-10

**Table S6:** GDM medicine number and weeks of treatment by the early management and usual care groups for complete case participants……………………………………………………………………………………...p-11

**Table S7:** Sensitivity analysis (two-sample t test and Wilcoxon ran-sum test) for the mean healthcare cost (SE) and mean cost differences (95% CI) per participant by cost category for the early management and usual care groups**.**………………………………...………………………….…………….………………………………p-12

**Table S8:** CHEERS Checklist 2020….………………………….…………….………………………………p-13

**References**……………………………………………………………………………………………………..p-15

**Figure S1: CONSORT diagram of screening, randomization, and follow-up of the TOBOGM trial.**


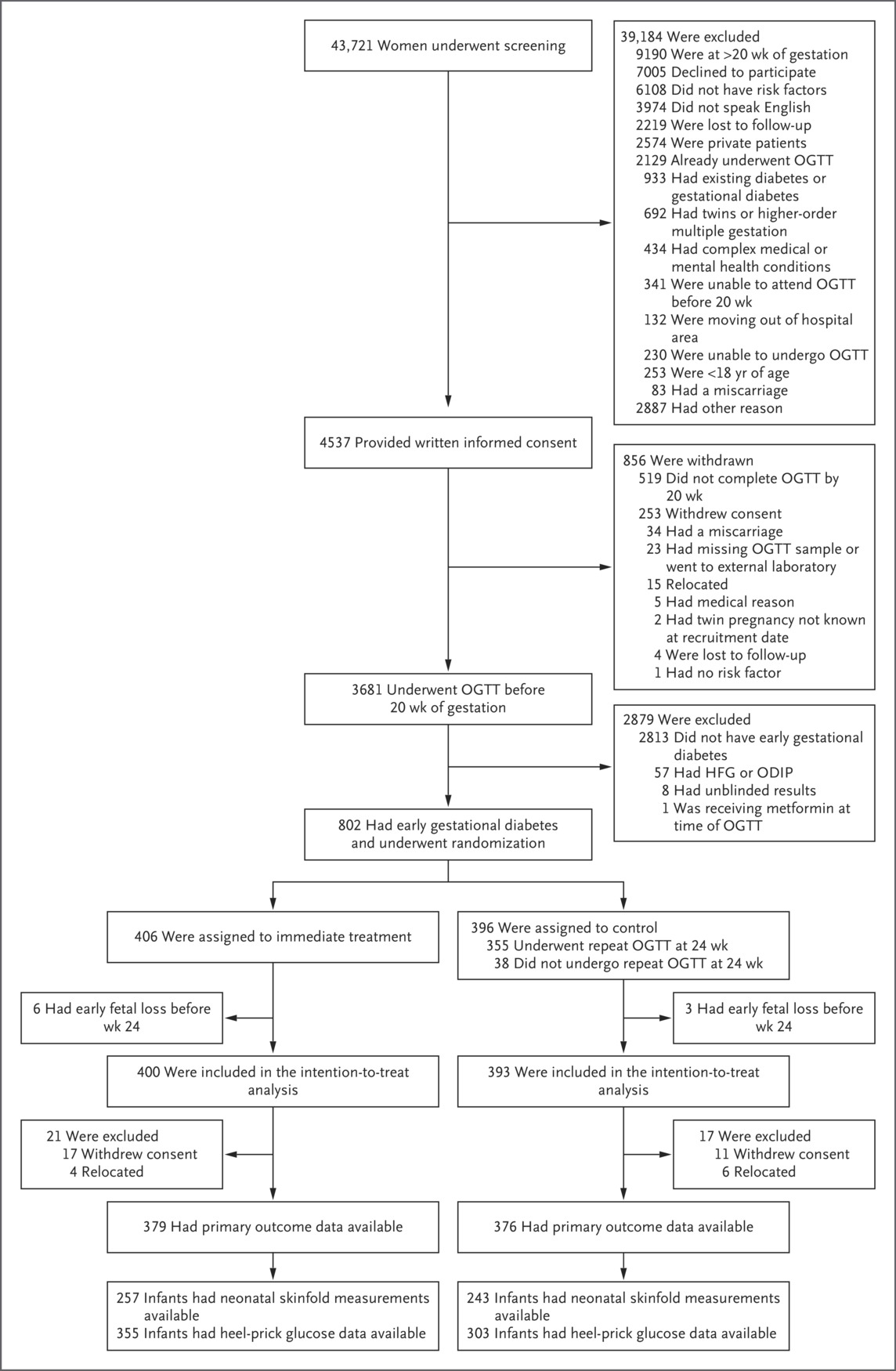


Source: Simmons D et al. The New England Journal of Medicine. 2023; 388(23):2132-2144.

*Abbreviations: HFG High fasting glucose; ODIP Overt diabetes in pregnancy; OGTT Oral glucose tolerance test.*

**Attachment S1 Cost estimation for early gestational diabetes mellitus (GDM) diagnosis:**

The total number of women screened for the randomized controlled trial (i.e. 43,271) included women with pre-existing type 1 and type 2 diabetes, or who already had been tested, and therefore was not used to estimate the cost for early GDM diagnosis; the number of women eligible for the trial (i.e. 3,681) were considered in estimating this cost. Of these, women that were randomized for the trial (i.e. 802), and those with GDM detected but excluded from the trial, including those with high fasting glucose or diabetes in pregnancy (i.e. 57), those with GDM unblinded results (i.e. 8), and those receiving metformin at time of oral glucose tolerance test (OGTT) (i.e. 1), totalling 868 participants would have had an OGTT at 24-28 weeks. Hence, the extra test was done only in those who did not have early GDM, i.e. 2813 (868 deducted from 3,681). Therefore, 3.2 (2813 divided by 868) women were tested, and the same number of OGTT was performed to identify one case of early GDM (not to be confused with the screening test, as screening was performed through the review of GDM risk factors). The cost of these additional 3.2 OGTT for each woman with early GDM identified was included as the cost of early GDM diagnosis in the early management group but not in the usual care group.

**Attachment S2: Missing data and multiple imputation**

Missing data was an important consideration in the analysis. In the 793 final sample, complete healthcare cost data were available for 742 (93.6%) participants. Missing data were examined using logistic regression and were not found to be associated with: smoking status, body mass index (BMI), age, primigravida, history of previous gestational diabetes (GDM), University qualification. Following careful consideration of the pattern of missing cost data, we proceeded with the assumption that the data were missing at random. Multiple imputation by chained equations was undertaken to impute missing values as the most suitable option for our missing data patterns.^2^ The MI procedure was performed using the MI command in Stata 17.0. The variables applied in the chained equations of imputation models were pragmatically chosen that included: age, primigravida, history of previous GDM, treatment arm and study centre. Ten complete data sets were generated for each set of missing data. Pooled estimates of costs were calculated from the generated datasets using Rubin’s rules.^3^

**Table S1: Unit cost of primary, secondary, and allied healthcare services utilized in the main analysis.**

| **Health care service** | **Source** | **Mean cost per unit (as of 1 July 2022)** | **Data source** |
| --- | --- | --- | --- |
| General practitioner | MBS item no. 23 Standard consultation (duration <20 minutes) | $28.01 | MBS |
| Endocrinologist | MBS item no. 110 Initial visit  MBS Item no. 116 Follow-up visit | $114.09   $57.12 | MBS |
| Obstetrician/Maternal-fetal medicine specialist | MBS item no. 16500 Antenatal attendance | $35.13 | MBS |
| Midwife | MBS item no. 82100 Initial antenatal attendance (duration >40 minutes)  MBS item no. 82105 Short subsequent antenatal attendance (duration <40 minutes) | $39.78     $24.07 | MBS |
| Dietician | MBS item no. 81120 Assessment for group services  MBS item no. 81125 Group services | $59.51    $14.83 | MBS |
| Diabetes educator | MBS item no. 81100 Assessment for group services  MBS item no. 81105 Group services | $59.51    $14.83 | MBS |
| Ultrasound | MBS item no. 55712, 55721 | $84.32 | MBS |
| Fetal non-stress test | MBS item no. 16514 Antenatal cardiotocography | $27.31 | MBS |
| Blood test | MBS item no. 66548 Oral glucose tolerance test (OGTT) in pregnancy for gestational diabetes mellitus | $14.02 | MBS |
| Emergency department | Weighted average of two AR-DRG cost weights relating to antenatal and other obstetric conditions - E1420A (Antenatal and other obstetric conditions, *complexity level A*), and E1420B (Antenatal and other obstetric conditions, *complexity level B*). | $387.81 (1 July 2019) $428.14 (1 July 2022 - adjusted for inflation) | IHACPA NHCDC Round 24  v:11 2019-20 |
| Maternal hospital admission | Weighted average of three AR-DRG cost weights relating to antenatal and other obstetric admissions - O66A (Antenatal and other obstetric admissions, *major complexity*)*,* O66B (Antenatal and other obstetric admissions, *intermediate complexity*), and O66C (Antenatal and other obstetric admissions, *minor complexity*). | $1,319.45 (1 July 2019) $1456.68 (1 July 2022 - adjusted for inflation) | IHACPA NHCDC Round 24  v:11 2019-20 |
| Birth by cesarean section | Weighted average of three AR-DRG cost weights relating to cesarean delivery - O01A (Cesarean delivery, *major complexity*), O01B (Caesarean delivery, *intermediate complexity*), and O01C (Cesarean delivery, *minor complexity*). | $9,507.37 (1 July 2019) $10,496.19 (1 July 2022 - adjusted for inflation) | IHACPA NHCDC Round 24  v:11 2019-20 |
| Vaginal birth (with 3^rd^/4^th^ degree tear) | Weighted average of two AR-DRG cost weights relating to vaginal delivery – O02A (Vaginal delivery w GIs, *major complexity*), and O02B (Vaginal delivery w GIs, *minor complexity*). | $8,344.05 (1 July 2019) $9,211.87 (1 July 2022 - adjusted for inflation) | IHACPA NHCDC Round 24  v:11 2019-20 |
| Vaginal birth (with instruments) | AR-DRG cost weight relating to 060B (Vaginal delivery, *intermediate complexity*) | $4,904.86 (1 July 2019) $5,415 (1 July 2022 - adjusted for inflation) | IHACPA NHCDC Round 24  v:11 2019-20 |
| Vaginal birth (without instruments) | AR-DRG cost weight relating to 060C (Vaginal delivery, *minor complexity*) | $3,556.73 (1 July 2019) $3,926.65 (1 July 2022 - adjusted for inflation) | IHACPA NHCDC Round 24  v:11 2019-20 |
| Neonatal intensive care unit (NICU) admission | Weighted average of ten AR-DRG cost weights relating to neonatal acute admissions: P03A, P03B, P04A, P04B, P05A, P05B, P06A, P06B, P07Z, P08Z. | $1,962.81/day (1 July 2019) $2,166.94/day (1 July 2022 - adjusted for inflation) | IHACPA NHCDC Round 24  v:11 2019-20 |
| Special care nursery (SCN) admission | Weighted average of twenty-three AR-DRG cost weights relating to neonatal acute admissions: P61Z, P62A, P62B, P63A, P63B, P64A, P64B, P65A, P65B, P65C, P65D, P66A, P66B, P66C, P66D, P67A, P67B, P67C, P67D, P68A, P68B, P68C, P68D. | $1,565.27/day (1 July 2019) $1,728.06/day (1 July 2022 - adjusted for inflation) | IHACPA NHCDC Round 24  v:11 2019-20 |
| Anesthesiologist | MBS item no. 17615, 17645, 17680 | $64.70 | MBS |
| Birth/Day/Women’s assessment unit | Non-admitted care Tier 2 4028 Midwifery & maternity | $147.29 (1 July 2019) $162.60 (1 July 2022 - adjusted for inflation) | IHACPA NHCDC Round 24  v:11 2019-20 |
| Pre-admission | Non-admitted care Tier 2 4007 Pre-admission & pre-anaesthesia | $176.18 (1 July 2019) $194.50 (1 July 2022 - adjusted for inflation) | IHACPA NHCDC Round 24  v:11 2019-20 |
| Consultant physician (cardiologist, hematologist, neurologist, ophthalmologist, orthopedic, pediatrician, urologist, dental, renal, thyroid) | MBS item no. 110 Initial attendance  MBS item no. 116 Review attendance | $114.09   $57.12 | MBS |
| Psychologist | MBS item no. 81000 Pregnancy support counselling | $54.47 | MBS |
| Podiatrist | MBS item no. 10962 | $46.40 | MBS |
| Physiotherapist | MBS item no. 10960 Allied health service | $46.40 | MBS |
| Mental health service | MBS item no. 10956 | $46.40 | MBS |
| Social worker | MBS item no. 81005 Pregnancy support counselling | $54.47 | MBS |
| Pre-eclampsia day stay | Non-admitted care Tier 2 4028 Midwifery & maternity | $147.29 (1 July 2019) $162.60 (1 July 2022 - adjusted for inflation) | IHACPA NHCDC Round 24 v:11 2019-20 |
| Nurse (mental health) | MBS item no. 81010 Pregnancy support counselling | $54.47 | MBS |
| Nurse (chronic disease) | MBS item no. 10997 | $8.95 | MBS |
| Blood glucose test strip |  | $10.71 (100 strip) | NDSS |

All costs were reported in US dollar ($) (adjusted for purchasing power parity 1.419 A$: 1 US$, 1 July 2022).
*Abbreviations: AR-DRG Australian Refined Diagnosis-Related Groups; IHACPA Independent Health and Aged Care Pricing Authority; MBS Medicare Benefits Schedule; NDSS National Diabetes Service Scheme; NHCDC National Hospital Cost Data Collection.*

**Table S2: Unit of healthcare service utilization per participant by cost category for the early management and usual care groups for complete case participants.**

| **Cost category** | **Early management group (n = 376) Mean (SE)** | **Usual care group (n = 366) Mean (SE)** |
| --- | --- | --- |
| General practitioner | 1.13 (0.11) | 1.13 (0.13) |
| Endocrinologist | 4.22 (0.29) | 1.67 (0.15) |
| Obstetrician/Maternal-fetal medicine specialist | 10.31 (0.32) | 7.86 (0.29) |
| Midwife | 5.01 (0.23) | 6.22 (0.27) |
| Miscellaneous healthcare providers^a^ | 1.56 (0.17) | 1.70 (0.19) |
| Dietician | 1.90 (0.10) | 1.34 (0.10) |
| Diabetes educator | 7.54 (0.32) | 3.65 (0.22) |
| Ultrasound | 2.90 (0.15) | 2.54 (0.12) |
| Fetal non-stress test | 0.02 (0.01) | 0.01 (0.005) |
| Blood test | 1.63 (0.11) | 1.51 (0.15) |
| Emergency department | 0.26 (0.05) | 0.17 (0.03) |
| Maternal hospital admission | 1.00 (0.02) | 0.98 (0.02) |

^a^ Include anesthesiologist, birth/day/women’s assessment unit, cardiologist, chronic disease nurse, consultant physician (dental, renal, thyroid), hematologist, mental health nurse, mental health service, neurologist, ophthalmologist, orthopedic, pediatrician, physiotherapist, podiatrist, pre-admission, preeclampsia day stay, psychologist, social worker, and urologist.
*Abbreviation: SE Standard error*.

**Table S3: Number and percentage of NICU and SCN admissions by the early management and usual care groups for complete case participants.**

|  | **Early management group (n = 376) N (%)** | **Usual care group (n = 366) N (%)** | **Total (n = 742) N (%)** |
| --- | --- | --- | --- |
| NICU admission | 9 (2.4) | 16 (4.4) | 25 (3.4) |
| SCN admission | 83 (22.1) | 85 (23.2) | 168 (22.6) |

*Abbreviations: % percentage; N Number; NICU Neonatal intensive care unit; SCN Special care nursery.*

**Table S4: Length of stay of NICU and SCN admissions by the early management and usual care groups for complete case participants.**

|  | **Early management group (n = 376) Number of days (mean) [SD]** | **Usual care group (n = 366) Number of days (mean) [SD]** | **Total (n = 742) Number of days (mean) [SD]** |
| --- | --- | --- | --- |
| NICU length of stay | 141.67 (0.38) [4.74] | 378.83 (1.04) [8.14] | 520.5 (0.70) [6.64] |
| SCN length of stay | 328.77 (0.87) [3.06] | 387.49 (1.06) [3.47] | 716.26 (0.97) [3.27] |

*Abbreviations: NICU Neonatal intensive care unit; SCN Special care nursery; SD Standard deviation.*

**Table S5: Number and percentage of mode of birth by the early management and usual care groups for complete case participants.**

|  | **Early management group (n = 376) N (%)** | **Usual care group (n = 366) N (%)** | **Total (n = 742) N (%)** |
| --- | --- | --- | --- |
| Vaginal birth without instruments | 201 (53.5) | 173 (47.3) | 374 (50.4) |
| Vaginal birth with instruments | 28 (7.5) | 34 (9.3) | 62 (8.4) |
| Vaginal birth with 3^rd^/4^th^ degree tear | 3 (0.8) | 13 (3.6) | 16 (2.2) |
| Cesarean section | 144 (38.3) | 146 (39.9) | 290 (39.1) |

*Abbreviations: % percentage; N Number.*

**Table S6: GDM medicine number and weeks of treatment by the early management and usual care groups for complete case participants.**

|  | **Early management group (n = 376) N** | **Usual care group (n = 366) N** | **Total (n = 742) N** |
| --- | --- | --- | --- |
| Insulin only | 165 | 130 | 295 |
| Metformin only | 35 | 16 | 51 |
| Both insulin and metformin | 54 | 22 | 76 |
|  | **Weeks of treatment Mean (SE) [95%CI]** | |  |
| Insulin only | 12.2 (0.8) [10.7, 13.7] | 5.0 (0.4) [4.2, 5.8] | 8.4 (0.5) [7.5, 9.2] |
| Metformin only | 18.8 (0.7) [16.3, 21.3] | 6.4 (0.3) [5.6, 7.2] | 12.7 (0.6) [11.2, 14.2] |
| Both insulin and metformin | 15.6 (0.8) [14.1, 17.0] | 5.6 (0.4) [4.8, 6.4] | 10.6 (0.5) [9.7, 11.6] |

*Abbreviations: CI Confidence interval; GDM Gestational diabetes mellitus; N Number; SE Standard error.*

**Table S7: Sensitivity analysis (two-sample t test and Wilcoxon ran-sum test) for the mean healthcare cost (SE) and mean cost differences (95% CI) per participant by cost category for the early management and usual care groups.**

| **Cost category** | **Early management group (n = 400) $ (SE)** | **Usual care group (n = 393) $ (SE)** | **Cost difference $ (95%CI)** | **Wilcoxon rank-sum (Mann-Whitney) test**  **p-value** |
| --- | --- | --- | --- | --- |
| Early GDM diagnosis approach^a^ | 54.92 (0.43) | 0 | **54.92 (51.38, 58.46)** | **<0.001** |
| Self-monitoring of blood-glucose | 98.11 (0.68) | 55.44 (2.45) | **42.68 (37.67, 47.69)** | **<0.001** |
| General practitioner | 44.88 (4.22) | 44.79 (4.74) | 0.09 (-12.35, 12.54) | 0.82 |
| Endocrinologist | 304.0 (19.97) | 129.14 (10.28) | **174.86 (130.76, 218.97)** | **<0.001** |
| Obstetrician/Maternal-fetal medicine specialist | 501.65 (15.84) | 392.22 (14.36) | **109.43 (67.46, 151.39)** | **<0.001** |
| Midwife | 163.97 (6.45) | 196.0 (7.32) | **-32.03 (-51.17, -12.88)** | **<0.001** |
| Miscellaneous healthcare providers^b^ | 215.66 (23.96) | 224.40 (24.69) | -8.74 (-76.28, 58.79) | 0.81 |
| Dietician | 79.17 (2.24) | 58.58 (2.51) | **20.59 (13.98, 27.19)** | **<0.001** |
| Diabetes educator | 181.81 (5.65) | 101.31 (4.60) | **80.49 (66.19, 94.80)** | **<0.001** |
| Ultrasound | 290.44 (14.09) | 255.05 (11.89) | 35.39 (-0.82, 71.59) | 0.07 |
| Fetal non-stress test | 0.77 (0.34) | 0.28 (0.14) | 0.49 (-0.23, 1.21) | 0.81 |
| Blood test | 27.24 (1.83) | 25.37 (2.45) | 1.87 (-4.13, 7.87) | 0.07 |
| Emergency department | 154.61 (27.26) | 98.81 (16.16) | 55.80 (-6.43, 118.03) | 0.51 |
| Maternal hospital admission | 2,081.21 (47.13) | 2,039.27 (47.38) | 41.94 (-89.24, 173.13) | 0.48 |
| Delivery of birth | 9,367.26 (215.78) | 9,726.20 (216.29) | -358.94 (-958.66, 240.77) | 0.22 |
| Neonatal intensive care unit | 1,219.59 (706.71) | 3,102.43 (1,218.42) | -1,882.83  (-4,648.84, 883.17) | 0.27 |
| Special care nursery | 2,158.93 (364.30) | 2,569.85 (414.14) | -410.92  (-1,493.66, 671.82) | 0.61 |
| Medication | 254.90 (15.56) | 128.73 (9.61) | **126.17 (90.27, 162.07)** | **<0.001** |
| Total healthcare | 17,195.49 (832.43) | 19,129.40 (1,322.06) | -1,933.91  (-5,001.56, 1,133.75) | 0.24 |

^a^ Appendix A. Supplementary data: Attachment S1.

^b^Include anesthesiologist, birth/day/women’s assessment unit, cardiologist, chronic disease nurse, consultant physician (dental, renal, thyroid), hematologist, mental health nurse, mental health service, neurologist, ophthalmologist, orthopedic, pediatrician, physiotherapist, podiatrist, pre-admission, preeclampsia day stay, psychologist, social worker, and urologist.
Statistical significance denoted by bold highlight.
*Abbreviations: $ United States dollar; 95%CI 95% Confidence interval; GDM Gestational diabetes mellitus; SE Standard error.*

**Table S8: CHEERS 2022 Checklist**

|  | **Item** | **Guidance for Reporting** | **Reported in section** |
| --- | --- | --- | --- |
| **TITLE** | | |  |
| Title | 1 | Identify the study as an economic evaluation and specify the interventions being compared. | Page 1 |
| **ABSTRACT** | | |  |
| Abstract | 2 | Provide a structured summary that highlights context, key methods, results and alternative analyses. | Page 2-3 |
| **INTRODUCTION** | | |  |
| Background and objectives | 3 | Give the context for the study, the study question and its practical relevance for decision making in policy or practice. | Page 4 |
| **METHODS** | | |  |
| Health economic  analysis plan | 4 | Indicate whether a health economic analysis plan was developed and where available. | Page 4 (Supplementary Figure S1) |
| Study population | 5 | Describe characteristics of the study population (such as age range, demographics, socioeconomic, or clinical characteristics). | Page 4 |
| Setting and location | 6 | Provide relevant contextual information that may influence findings. | Page 4, 5 |
| Comparators | 7 | Describe the interventions or strategies being compared and why chosen. | Page 4-6 |
| Perspective | 8 | State the perspective(s) adopted by the study and why chosen. | Page 4, 5 |
| Time horizon | 9 | State the time horizon for the study and why appropriate. | Page 5, 6 |
| Discount rate | 10 | Report the discount rate(s) and reason chosen. | Page 6 |
| Selection of outcomes | 11 | Describe what outcomes were used as the measure(s) of benefit(s) and harm(s). | Page 5 |
| Measurement of outcomes | 12 | Describe how outcomes used to capture benefit(s) and harm(s) were measured. | Page 5 |
| Valuation of outcomes | 13 | Describe the population and methods used to measure and value outcomes. | Page 4, 5 |
| Measurement and valuation of resources  and costs | 14 | Describe how costs were valued. | Page 5, 6 |
| Currency, price date, and conversion | 15 | Report the dates of the estimated resource quantities and unit costs, plus the currency and year of conversion. | Page 5, 6 (Supplementary Table S2) |
| Rationale and  description of model | 16 | If modelling is used, describe in detail and why used. Report if the model is publicly available and where it can be accessed. | NA |
| Analytics and assumptions | 17 | Describe any methods for analysing or statistically transforming data, any extrapolation methods, and approaches for validating any model used. | NA |
| Characterizing heterogeneity | 18 | Describe any methods used for estimating how the results of the study vary for sub-groups. | Page 6 |
| Characterizing  distributional effects | 19 | Describe how impacts are distributed across different individuals  or adjustments made to reflect priority populations. | NA |
| Characterizing uncertainty | 20 | Describe methods to characterize any sources of uncertainty in the analysis. | Page 6 |
| Approach to engagement with patients and others affected by the study | 21 | Describe any approaches to engage patients or service recipients, the general public, communities, or stakeholders (e.g., clinicians or payers) in the design of the study. | NA |
| **RESULTS** | | |  |
| Study parameters | 22 | Report all analytic inputs (e.g., values, ranges, references) including uncertainty or distributional assumptions. | Page 7 (Supplementary Table S1) |
| Summary of main results | 23 | Report the mean values for the main categories of costs and outcomes of interest and summarise them in the most appropriate overall measure. | Page 7, 8 (Table 1, 2 and Figure 1, 2) |
| Effect of uncertainty | 24 | Describe how uncertainty about analytic judgments, inputs, or projections affect findings. Report the effect of choice of discount rate and time horizon, if applicable. | Page 7, 8 (Table 2, Figure 2) |
| Effect of engagement with patients and others affected by the study | 25 | Report on any difference patient/service recipient, general public, community, or stakeholder involvement made to the approach or findings of the study | NA |
| **DISCUSSION** | | |  |
| Study findings, limitations, generalizability, and current knowledge | 26 | Report key findings, limitations, ethical or equity considerations not captured, and how these could impact patients, policy, or practice. | Page 8, 9 |
| **OTHER RELEVANT INFORMATION** | | | |
| Source of funding | 27 | Describe how the study was funded and any role of the funder in the identification, design, conduct, and reporting of the analysis | Page 2, 6, 10 |
| Conflicts of interest | 28 | Report authors conflicts of interest according to journal or  International Committee of Medical Journal Editors requirements. | Page 10 |

Husereau D, Drummond M, Augustovski F, de Bekker-Grob E, Briggs AH, Carswell C, Caulley L, Chaiyakunapruk N, Greenberg D, Loder E, Mauskopf J, Mullins CD, Petrou S, Pwu RF, Staniszewska S; CHEERS 2022 ISPOR Good Research Practices Task Force. Consolidated Health Economic Evaluation Reporting Standards 2022 (CHEERS 2022) Statement: Updated Reporting Guidance for Health Economic Evaluations. BMJ. 2022;376:e067975. The checklist is Open Access distributed in accordance with the terms of the Creative Commons Attribution (CC BY 4.0) license, which permits others to distribute, remix, adapt and build upon this work, for commercial use, provided the original work is properly cited. See: [http://creativecommons.org/licenses/by/4.0/.](http://creativecommons.org/licenses/by/4.0/)

**References:**

1. Simmons D, Immanuel J, Hague WM, et al. Treatment of Gestational Diabetes Mellitus Diagnosed Early in Pregnancy. The New England Journal of Medicine. 2023;388(23):2132-2144.
2. Faria R, Gomes M, Epstein D, et al. A guide to handling missing data in cost-effectiveness analysis conducted within randomised controlled trials. *Pharmacoeconomics*. 2014; 32:1157–70.
3. Rubin DB. Multiple Imputation for Nonresponse in Surveys. New York: John Wiley & Sons; 2004.
